# Effect of Silver Diamine Fluoride Application Methods on Dentin Microhardness and Durability Under pH Cycling: An In Vitro Study

**DOI:** 10.1002/cre2.70190

**Published:** 2025-07-31

**Authors:** Farideh Darabi, Parmida Farzam, Mehrsima Ghavami‐Lahiji

**Affiliations:** ^1^ Department of Restorative Dentistry, Dental Sciences Research Center, School of Dentistry Guilan University of Medical Sciences Rasht Iran; ^2^ Dental Sciences Research Center, School of Dentistry Guilan University of Medical Sciences Rasht Iran

**Keywords:** acid challenge, administration, dentin, hardness, In Vitro techniques, silver diamine fluoride, topical

## Abstract

**Objectives:**

This study investigated the effects of various application methods of silver diamine fluoride (SDF) on dentin microhardness and its durability under a 14‐day acid challenge. The primary goal was to identify an optimal conservative SDF application protocol for clinical dentistry.

**Material and Methods:**

Mid‐coronal dentin disc samples from human premolars were prepared through two horizontal cuts and polished. Samples were randomly divided into three groups of ten: (1) SDF‐NR (SDF applied for 3 min, excess removed with cotton); (2) SDF‐R‐R (SDF applied, rinsed, and polished after 24 h); (3) SDF‐R (SDF applied for 3 min, then rinsed). Vickers microhardness was measured at three stages: initial, post‐demineralization, and post‐SDF application. In the second phase, a control group (no SDF) and a test group (SDF‐R) underwent a 14‐day pH‐cycling regimen, with final hardness changes evaluated at a significance level of 0.05.

**Results:**

Significant microhardness increases were observed in all groups following SDF application compared to demineralized conditions (*p* = 0.001). Although the decrease in microhardness after acid challenge in the SDF group was less than in the control group, this difference was not statistically significant (*p* = 0.423).

**Conclusion:**

Different SDF application methods significantly enhance dentin microhardness post‐demineralization. Nevertheless, the acid challenge revealed minimal differences between the control and test group, indicating that reapplication of SDF may be necessary for sustained effectiveness.

## 1 Introduction

Silver diamine fluoride (SDF) has emerged as a powerful agent in the paradigm shift from traditional “drill and fill” dentistry toward Minimal Intervention Dentistry (MID) (Nishino et al. 1969; Naryal et al. 2025). Numerous randomized controlled trials and systematic reviews have demonstrated SDF's effectiveness in arresting dental caries, particularly in primary teeth and permanent root caries (Vollú et al. 2019; Amorim et al. 2025; Rogalnikovaitė et al. 2024; Boobalan et al. 2025). Its primary mechanism, potent antimicrobial activity against cariogenic biofilms, has been well documented, with strong inhibitory effects on key oral pathogens (Fakhruddin et al. 2020; Mei et al. 2013; Alowid et al. 2024). Additionally, SDF is cytocompatible, promotes remineralization of enamel and dentin, prevents collagen degradation, and can reduce dentin hypersensitivity (Oliveira et al. 2018; García‐Bernal et al. 2022). These properties have led to the integration of SDF into dental curricula and public health policies in several countries, underscoring its value in minimally invasive care (Zheng et al. 2022; Zhang et al. 2024).

Despite these advantages, the widespread clinical adoption of SDF remains limited (Horst et al. 2016). The most significant barrier is the black discoloration of carious lesions arrested by SDF, which is particularly problematic for patients and caregivers when anterior teeth are involved. This staining, caused by silver phosphate deposition, substantially reduces patient acceptance (Seifo et al. 2020; Crystal et al. 2017). Additionally, there is a lack of consensus on optimal application protocols, such as whether to rinse after application, and concerns remain about potential soft tissue irritation and compromised bonding of restorative materials following SDF use (Uctasli et al. 2023; Yan et al. 2022).

To address these challenges and expand SDF's clinical utility, several innovative strategies have been proposed. The use of potassium iodide (KI) following SDF application has shown promise in reducing discoloration, as well as alternative protocols such as rinsing, resurfacing, and delayed restoration (Uctasli et al. 2023; Lutgen et al. 2018; Alsagob et al. 2022; Xu et al. 2024; Ricalde et al. 2025). Some countries have successfully integrated SDF into national oral health programs, highlighting the potential for broader policy adoption (El Azrak 2024; Gao et al. 2021). Laboratory studies continue to explore ways to optimize SDF's benefits while minimizing drawbacks, including improving the bond strength of restorative materials and addressing esthetic challenges (Lutgen et al. 2018).

However, these opportunities are tempered by ongoing challenges and limitations. There is still no universally accepted or standardized protocol for SDF use, and expert consensus remains divided on key procedural details (Horst et al. 2016; Yan et al. 2022). Some evidence suggests that certain modifications, which reduce staining, such as rinsing or using potassium iodide, may also diminish SDF's anticaries efficacy (Zhao et al. 2017; Detsomboonrat et al. 2022; Papagatsiou et al. 2023). Furthermore, most existing studies have focused on short‐term outcomes, and little is known about the durability of SDF's effects on dentin microhardness under dynamic, real‐world oral conditions, including pH fluctuations (Cifuentes‐Jiménez et al. 2023; Iovan et al. 2023). These uncertainties hinder the development of evidence‐based guidelines and limit the full integration of SDF into routine dental practice.

To date, the literature has not adequately addressed the long‐term effects of SDF on dentin microhardness under dynamic, clinically relevant conditions, nor has it provided clear guidance on how different application protocols influence these outcomes. The present study is novel in its comprehensive, comparative evaluation of multiple SDF application methods and their impact on dentin microhardness over a sustained period of pH cycling, which closely simulates the fluctuating oral environment. By systematically investigating both the immediate and durable effects of these protocols, this study fills a critical gap in the evidence base and offers actionable insights for optimizing SDF use in clinical practice. These findings have the potential to inform the development of standardized, evidence‐based application protocols, thereby advancing minimally invasive dentistry and improving patient care through more predictable and effective caries management.

Thus, the present in vitro study was conducted to evaluate how different methods of application affect dentin microhardness and the durability of SDF treatment in an in vitro pH cycling model over 14 days. The null hypotheses tested in this study were that (1) no significant differences in dentin microhardness would exist between different SDF application methods and that (2) the changes in dentin microhardness after in vitro pH cycling would not be significantly different in SDF‐treated and control samples.

## 2 Materials and Methods

This in vitro study was conducted following ethical approval obtained from Guilan University of Medical Sciences with the identification code IR.GUMS. REC.1403.031. To determine the required sample size, a power analysis was performed. With a significance level (α) of 5% and a desired power (1‐β) of 80%, the minimum sample size required for each group was calculated to be 9, using the following formula (Firouzmandi et al. 2020).

$$n1=n2=\frac{{\left(Z_{1-\frac{\alpha }{2}}+Z_{1-\beta }\right)}^{2}({\sigma^{2}}_{1}-{\sigma^{2}}_{2})}{{\left({µ}_{1}-{µ}_{2}\right)}^{2}}$$

To account for potential sample loss or variability, 10 extracted teeth were allocated to each of the four groups (*n* = 10 per group: one control and three experimental groups), resulting in a total of 40 teeth.

### 2.1 Tooth Selection and Preparation

Extracted human premolar teeth (*n* = 40) were randomly assigned to one of four experimental groups. Inclusion criteria were intact human premolars (teeth 4 or 5 in palmer notation system) extracted for surgical or orthodontic reasons, without any evidence of caries, cracks, or fractures. Teeth with pre‐existing restorations, fractures or cracks, severe anomalies or discoloration, and any visible carious lesions were excluded from the study.

Following extraction, all teeth were cleaned using manual scaling and brushing with a universal polishing paste (Ivoclar Vivadent, Schaan, Liechtenstein). For disinfection, the samples were stored in a 0.5% chloramine‐T solution at 4°C for 7 days to prevent bacterial growth (Iovan et al. 2023). Afterwards, the specimens were stored in artificial saliva to maintain hydration and simulate the oral environment during the experiment (Alexandria et al. 2017).

The occlusal enamel was removed by making a horizontal section using a precision saw (Low Speed Precision Cutter, Nemofanavaran Pars, Mashhad, Iran). Complete removal of the occlusal enamel was verified visually and ensured through uniform precision cuts. A second horizontal section was made at the approximate level of the pulp chamber with the precision saw to remove the coronal portion of the tooth to expose a flat dentin surface. The resulting dentin discs were then embedded in self‐curing acrylic resin blocks. Dentin surfaces were then sequentially wet‐ground using silicon carbide abrasive papers of increasing grit size (coarse to fine) to produce a flat and smooth surface. Samples were then cleaned in an ultrasonic bath for 10 min to remove any polishing debris. Each dentin surface was examined under a light microscope at 40x magnification to ensure they were smooth and free of scratches. The samples were then randomly assigned to four groups using a simple randomization technique. They were marked and tracked throughout all experimental stages to monitor changes over time.

#### 2.1.1 Phase 1: Different SDF Application Methods and Their Effect on Hardness

##### 2.1.1.1 Baseline Microhardness Measurement

The baseline microhardness of each sample was determined using a Vickers microhardness tester (Baresiss, Oberdischingen, Germany). For the Vickers surface microhardness measurements, samples from each group underwent indentation with a Vickers diamond indenter on the microhardness testing machine. A load of 50 gf was applied for a dwell time of 15 s, and three indentations were made on the polished dentin surface of each sample. The mean value was calculated and recorded as the surface microhardness for that specimen. Microhardness values were expressed as Vickers Hardness Number (VHN), and reported in kgf/mm².

##### 2.1.1.2 Demineralization Protocol

Following baseline microhardness measurements, the teeth were demineralized according to a previously published protocol (Marquezan et al. 2009). The samples were immersed in a demineralizing solution containing 2.2 mM calcium chloride (CaCl_2_), 2.2 mM sodium dihydrogen phosphate (NaH_2_PO_4_), and 50 mM acetic acid, adjusted to a pH of 5.0. The samples were incubated in this solution for 2 h. After demineralization, a second microhardness measurement (*H*_d_) was taken (Buzalaf et al. 2010; Memis Ozgul et al. 2015).

- *H*_b_ = Baseline microhardness
- *H*_d_ = Microhardness after demineralization

##### 2.1.1.3 Treatment Groups

**Figure 1:**
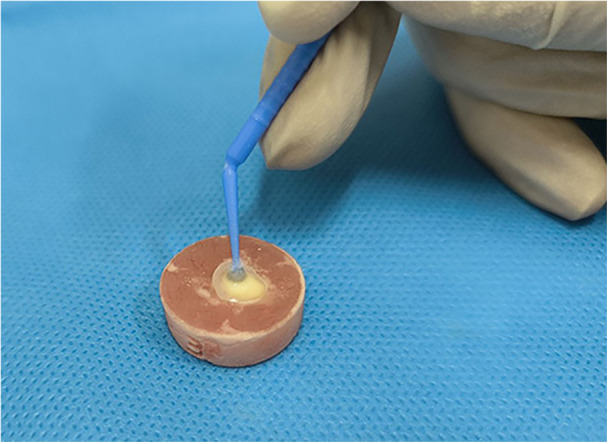
Application of SDF on dentin.

The samples were randomly divided into the following treatment groups (*n* = 10 per group):

1. **SDF‐No Rinse (SDF‐NR):** Samples in this group were treated with a 38% SDF solution (Knockout, Tuffbites, Jhajjar, Haryana, India) for 1 min (Figure 1). The samples were then kept at room temperature for 3 min. Afterwards, excess material was gently removed with a cotton pellet without rinsing. A third microhardness measurement (*H*_a_) was then performed.
  - *H*_a_ = Microhardness after SDF application.
2. **SDF‐Rinse‐Resurface (SDF‐R‐R):** Samples in this group were treated with a 38% SDF solution for 1 min, followed by isolation at room temperature for 3 min. The samples were then rinsed with distilled water for 30 s. After 24 h, the surfaces were resurfaced using 50 cycles of abrasion with 600‐grit sandpaper in a figure‐8 motion. A third microhardness measurement (Ha) was then performed (Lutgen et al. 2018).
3. **SDF‐Rinse (SDF‐R):** Samples in this group were treated with a 38% SDF solution for 1 min, followed by isolation at room temperature for 3 min. The samples were then rinsed with distilled water for 30 s. A third microhardness measurement (*H*_a_) was then performed (Lutgen et al. 2018).

##### 2.1.1.4 Microhardness Analysis and Statistical Evaluation

Hardness changes due to demineralization (*H*_d_ – *H*_b_), hardness changes after SDF treatment (*H*_a_ – *H*_d_), and relative changes [(*H*_a_ – *H*_d_)/*H*_d_] were calculated and subjected to statistical analysis to determine the effects of demineralization and subsequent SDF treatment.

#### 2.1.2 Phase 2: Long‐Term Effect of SDF Under pH Cycling

To simulate the clinical oral environment and assess the long‐term effects of SDF treatment under fluctuating pH conditions, a pH‐cycling regimen was implemented. Since potassium iodide was not used in this study to control discoloration, a clinically relevant SDF application and rinsing protocol were used in this phase. Initially, baseline microhardness (*H*_b_) and post‐demineralization microhardness (*H*_d_) were recorded for all samples, as described earlier:

1. **Control Group:** This group did not receive SDF treatment but was subjected to pH cycling after demineralization. Samples were immersed in a demineralizing solution (pH 5.0) for 2 h, followed by immersion in a remineralizing solution (1.5 mM calcium chloride, 0.9 mM sodium dihydrogen phosphate, and 0.15 M potassium chloride (KCl), pH 7.0) for 22 h (Buzalaf et al. 2010; Memis Ozgul et al. 2015). This cycle was repeated daily for 2 weeks. After 2 weeks of pH cycling, the final microhardness (*H*_f_) was measured.

- *H*_f_ = Final microhardness after pH cycling.

2. **Experimental Group:** The samples from the SDF‐R group in Phase 1 were then subjected to the same pH‐cycling regimen as the control group (2 h in demineralizing solution, 22 h in remineralizing solution, repeated daily for 2 weeks). After 2 weeks of pH cycling, the final microhardness (*H*_f_) was measured.

The following parameters were assessed to evaluate the effects of demineralization, SDF treatment, and pH cycling, both with and without prior SDF application.

- *H*_d_ – *H*_b_: Change in microhardness due to demineralization.
- *H*_f_ – *H*_d_: Change in microhardness after pH cycling in both control and experimental groups.
- *H*_d_/*H*_f_: Ratio of microhardness after demineralization to final microhardness after pH cycling.

##### 2.1.2.1 Statistical Analysis

Statistical analysis was performed using IBM SPSS Statistics version 23. Normality and homogeneity of variances were assessed using the Shapiro–Wilk and Levene's tests, respectively. For two‐group comparisons, independent *t*‐tests were used. For multiple group comparisons, ANOVA with Tukey's post‐hoc test was applied. Repeated measures ANOVA was used for within‐group comparisons over time. The significance level was set at α = 0.05.

## 3 Results

**Figure 2:**
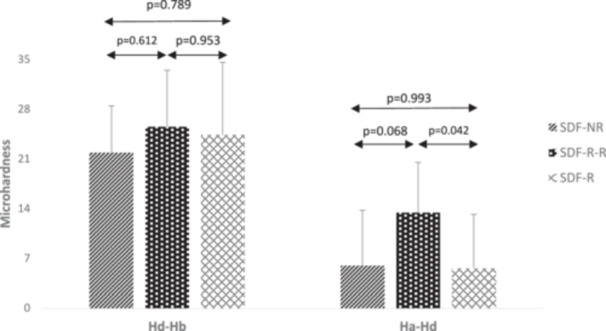
Microhardness differences of different SDF application methods at various time intervals (VHN, kgf/mm²).

Table 1 presents the mean microhardness values at different time intervals across the experimental groups. The microhardness loss after demineralization (*H*_d_ – *H*_b_) was reported for each group, and one‐way ANOVA indicated no statistically significant difference among the three groups (*p* = 0.626). The difference between microhardness after SDF application and after demineralization (*H*_a_ – *H*_d_) was also reported. One‐way ANOVA revealed a statistically significant difference among the three groups (*p* = 0.046), with the greatest change observed in the SDF‐R‐R group. Subsequently, Tukey's post hoc test showed no statistically significant difference between the SDF‐NR and SDF‐R groups (*p* = 0.993). However, pairwise comparisons between the SDF‐R and SDF‐R‐R groups revealed significant differences (*p* = 0.042) (Figure 2). Furthermore, the relative changes for each group were calculated and compared using one‐way ANOVA, which showed no statistically significant difference among the three groups (*p* = 0.175).

**Table 1:** Dentin microhardness alterations after different SDF application methods at various time intervals (VHN, kgf/mm²).

Groups	*H*_b_	*H*_d_	*H*_a_	*p*‐value*	*H*_d_ – *H*_b_ **	*H*_a_ – *H*_d_ **	Relative change**	*H*_a_/*H*_d_ **
SDF‐NR	72.27 ± 6.57	50.39 ± 8.78	56.39 ± 7.58	< 0.001	−21.88 ± 6.57	6.00 ± 7.77	0.14 ± 0.17	1.14 ± 0.17
SDF‐R‐R	73.01 ± 7.06	47.54 ± 6.54	60.97 ± 7.87	< 0.001	−25.47 ± 7.99	13.43 ± 7.07	0.29 ± 0.18	1.29 ± 0.18
SDF‐R	63.89 ± 6.50	39.53 ± 9.43	45.15 ± 9.50	< 0.001	−24.36 ± 10.23	5.63 ± 7.55	0.17 ± 0.22	1.17 ± 0.22
*p*‐value**					0.626	0.046	0.175	0.175

*H*_b_, baseline microhardness; *H*_d_, microhardness after demineralization; *H*_a_, microhardness after SDF application.

* Repeated measure.

** One‐way ANOVA test, Relative change: (*H*_a_ – *H*_d_)/*H*_d_.

To monitor changes in microhardness within each specimen across the different conditions over time, a repeated measures analysis was used. Repeated measures analysis revealed statistically significant changes in mean microhardness over time within each group (*p* < 0.001) (Table 1).

**Figure 3:**
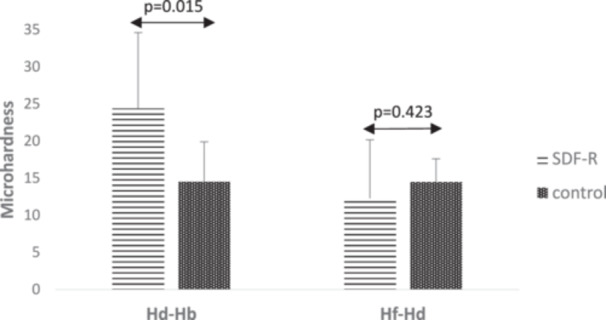
Microhardness differences of control and SDF‐treated specimens under pH cycling (VHN, kgf/mm²).

In Table 2, the mean microhardness values of the two groups, SDF‐R and control, are reported across different measurement time points. After 2 weeks of pH cycling, microhardness in the SDF‐treated group decreased by 12.28 ± 7.86 compared to post‐demineralization, while the control group, which received no treatment, exhibited a greater decrease (14.47 ± 3.11). Although the difference between the two groups was not statistically significant (*p* = 0.423), a tendency for less microhardness decrease in the SDF‐R group was noted. Additionally, the relative changes between the 2‐week posttreatment and post‐demineralization time were calculated for each group. Statistical analysis showed no significant difference in relative changes between the two groups (*p* = 0.680) (Figure 3).

**Table 2:** Dentin microhardness alterations in control and SDF‐treated specimens under pH Cycling (VHN, kgf/mm²).

Groups	*H*_b_	*H*_d_	*H*_f_	*p*‐value*	*H*_d_ – *H*_b_ **	*H*_f_ – *H_d_* **	Relative change**	*H*_f_/*H_d_* **
SDF‐R	63.89 ± 6.50	39.53 ± 9.43	27.25 ± 6.69	< 0.001	−24.36 ± 10.23	−12.28 ± 7.86	0.29 ± 0.15	0.70 ± 0.15
Control	60.06 ± 3.78	45.55 ± 2.55	31.08 ± 3.43	< 0.001	−14.51 ± 5.38	−14.47 ± 3.11	0.32 ± 0.07	0.68 ± 0.07
*p*‐value**					0.015	0.423	0.680	0.680

*H*_b_, baseline microhardness; *H*_d_, microhardness after demineralization; *H*_f_, final microhardness after pH cycling.

* Repeated measure.

** Independent *t*‐test, Relative change: (*H*_f_ – *H*_d_)/*H*_d_.

The results of repeated measures analysis revealed statistically significant changes in mean microhardness over time within each group (*p* < 0.001) (Table 2).

**Figure 4:**
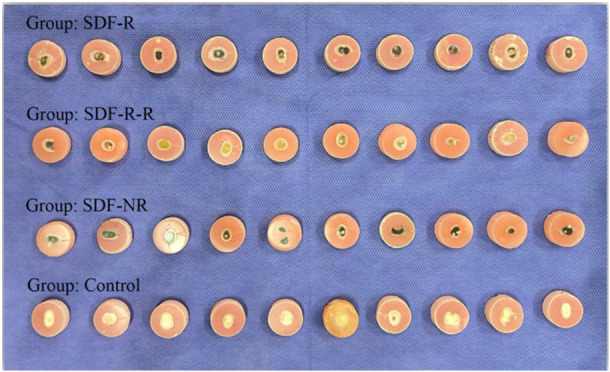
All specimens at the end of the experiment.

In Figure 4, all samples can be observed after the experiment. Although we did not directly evaluate color change, based on Figure 4, the results of SDF‐R‐R group seem promising in terms of appearance. The color change in the other two groups was similar.

## 4 Discussion

This study investigated the effects of various SDF application protocols on dentin microhardness across two distinct phases. Phase 1 evaluated three groups – SDF‐NR (no rinse), SDF‐R‐R (rinse and resurface), and SDF‐R (rinse) – to assess the immediate impact of different application methods on microhardness. Phase 2 examined the long‐term effects of SDF application by subjecting the control and SDF‐R groups to 2 weeks of pH cycling.

The findings from Phase 1 revealed that all three SDF groups exhibited a significant increase in microhardness following SDF application compared to the demineralized state. This supports the effectiveness of SDF in enhancing the microhardness of carious dentin and highlights its potential to promote remineralization and arrest caries progression. As demonstrated by Mei et al. the mechanism of SDF's caries‐arresting effect involves remineralization of the dentin surface through the deposition of fluoride, silver, and other ions, leading to the precipitation of mineral salts within dentinal tubules and the surface layer. The presence of silver ions contributes an antimicrobial effect, further preventing caries progression (Mei et al. 2018).

Consistent with previous studies (Rogalnikovaitė et al. 2024; Samani et al. 2024), our results support the use of SDF as a noninvasive and effective treatment for increasing carious dentin microhardness. Adjunctive approaches, such as light curing in combination with SDF application, have shown improved results in increasing dentin microhardness. However, this method may also lead to greater tooth discoloration due to increased silver deposition (Makwani et al. 2024).

The SDF‐R‐R group exhibited higher microhardness values compared to non‐resurfaced groups. This may be attributed to SDF penetration depths. Manuschai et al. reported SDF penetration ranging from 629 to 2516 μm after 3 min of application in deep caries lesions, as observed via Micro‐CT and Field emission scanning electron microscopy coupled with energy‐dispersive X‐ray spectroscopy (FESEM‐EDS) (Manuschai et al. 2021). In contrast, Crystal et al. noted approximately 600 μm of penetration after 1 min of application followed by rinsing (Crystal et al. 2023). Given the 3‐min application time used in our study, SDF penetration likely exceeded 600 μm.

Our 2‐h caries induction protocol using a pH of 5, based on the model by Zhang et al. would have produced lesions approximately 50 μm deep (Zhang et al. 2020). In addition, the resurfacing in the SDF‐R‐R group removed roughly 0.1 mm of superficial dentin (Lutgen et al. 2018). This would mean in this group, resurfacing effectively eliminated the weaker, superficial layer while retaining the deeper, silver‐reinforced dentin. This likely explains the increased microhardness observed in the SDF‐R‐R group compared to the others.

Lutgen et al. investigated the impact of SDF on the bond strength of adhesives to sound dentin and found that, while SDF does not inherently enhance bonding, rinsing and surface polishing can improve bond performance (Lutgen et al. 2018). Similarly, a study by Muniz et al. identified several mechanisms through which SDF may improve bond strength, including the formation of stable mineral compounds and protection of the collagen matrix. They reported that the formation of calcium fluoride and fluorapatite (with strengthened covalent bonds in an alkaline environment) helps preserve mineral content and increase dentin hardness, ultimately creating a stable and acid‐resistant substrate for bonding. By protecting collagen, SDF also preserves the structural integrity of dentin, resulting in stronger bonding (Muniz et al. 2024). However, Intajak et al. observed a reduction in bond strength in SDF‐treated dentin, possibly due to adverse interactions between adhesive components and SDF residues. Generally, etch‐and‐rinse adhesive systems tended to perform better than self‐etch systems when used with SDF‐treated dentin (Intajak et al. 2024).

The esthetic impact of SDF application remains a significant clinical concern (Seifo et al. 2020). Our SDF‐R‐R protocol, which included rinsing and resurfacing, showed promise in reducing esthetic drawbacks. This aligns with Alsagob et al.'s observation, who reported that delaying restoration following SDF application can reduce discoloration (Alsagob et al. 2022). Although discoloration was not quantitatively assessed in our study, visual inspection (Figure 4) suggests that the SDF‐R‐R protocol may better preserve the appearance of the treated tooth.

In this study, microhardness in SDF‐NR and SDF‐R groups did not significantly differ (Table 1). While rinsing, as observed by Lutgen et al. via SEM, may remove surface mineral deposits from SDF‐treated dentin, it does not prevent key ions from penetrating deeply. Therefore, despite superficial changes, rinsing appears to have minimal impact on overall microhardness. Furthermore, in clinical cases involving subsequent resin restoration, rinsing may improve bonding conditions (Lutgen et al. 2018).

The present study aimed to mimic oral conditions following SDF application using an alternating demineralization and remineralization (DEM and REM) regimen in Phase 2. To the best of the authors' knowledge, few studies have evaluated the effectiveness of the substance in this respect. The present study design differed from previous research in several key aspects (Firouzmandi et al. 2020; Srisomboon et al. 2021): (1) a pH‐cycling regimen was employed to better replicate the dynamic oral environment; (2) microhardness was monitored at multiple stages, providing a comprehensive view of changes over time; and (3) the acid challenge regimen closely mimicked clinical pH fluctuations, unlike some prior studies (Firouzmandi et al. 2020).

The results of Phase 2 showed that, although differences between stages were not statistically significant, the SDF‐R group exhibited less microhardness loss after pH cycling compared to the control group. This suggests that SDF provides some protection against demineralization, even under challenging conditions.

However, our findings, along with those of Iovan et al. indicate that the protective layer created by SDF (or SDF + KI) may not be sufficient to fully protect dentin when exposed to continuous acid attacks (Iovan et al. 2023). A reduction in microhardness following SDF application and pH cycling implies that the rate of demineralization exceeded the remineralization effect provided by SDF.

To improve the long‐term effect of SDF and increase dentin microhardness following pH cycling, several strategies could be considered. These include repeated SDF application, light curing, combination with other materials (e.g., fluoride‐containing products), and dietary modifications to minimize pH changes in the oral environment.

The concept of repeated SDF application is supported by several studies. Zaffarano et al. found SDF is effective in arresting cavitated lesions in primary molars, especially when applied biannually during the initial years (Zaffarano et al. 2022). Similarly, Horst et al. demonstrated that a weekly application protocol for 3 weeks, followed by an annual dose, was more effective than a single annual dose alone (Horst et al. 2016). These findings suggest that SDF should be integrated into an ongoing caries management strategy rather than employed as a one‐time treatment.

## 5 Limitations

This study has several limitations that should be considered when interpreting the results. First, the chemical caries model employed may not fully replicate the complex pathophysiology of natural caries formation, as it does not account for the dynamic interactions within the oral environment (e.g., biofilm activity and host factors). Second, the pH cycling duration was limited to 2 weeks, offering only short‐term insight into the durability of SDF effects and potentially failing to reflect long‐term clinical outcomes. Third, the in vitro design inherently lacks key clinical environmental factors such as salivary proteins, oral biofilm, and other biological variables that may influence the behavior and effectiveness of SDF in vivo. Future studies incorporating longer observation periods and more clinically relevant models are warranted to better simulate oral conditions and validate these findings.

Future research should prioritize evaluating the effects of repeated SDF applications under simulated oral conditions and developing strategies to enhance the durability of SDF effects, particularly in high‐risk patients. The long‐term clinical efficacy of SDF application methods should be considered.

## 6 Conclusion

This study demonstrated that SDF significantly increased dentin microhardness, regardless of the application method. Rinsing and resurfacing protocols did not compromise this benefit, supporting their use in addressing clinical concerns such as bonding and discoloration. However, the protective effect of a single SDF application diminished after repeated acid challenges, indicating that periodic reapplication may be necessary for sustained efficacy. By systematically comparing different SDF protocols under simulated cariogenic conditions, this study provides novel, evidence‐based guidance for optimizing minimally invasive caries management and highlights the clinical relevance of protocol selection.

## Author Contributions

**Farideh Darabi:** conceptualization, project administration, supervision. **Parmida Farzam:** methodology, investigation, interpretation of data, writing – original draft, visualization. **Mehrsima Ghavami‐Lahiji:** conceptualization, methodology, investigation, interpretation of data, writing – original draft, review and editing. All authors read and approved the final manuscript.

## Ethics Statement

This study was conducted in accordance with ethical standards and received approval from the Research Ethics Committee of Guilan University of Medical Sciences under the approval code IR.GUMS.REC.1403.031. The study utilized human premolars that were extracted for orthodontic reasons. The aforementioned Ethics Committee specifically approved the use of these extracted teeth.

## Conflicts of Interest

The authors declare no conflicts of interest.

## Data Availability

The data that support the findings of this study are available from the corresponding author upon reasonable request.
